# Heme binding to the SARS-CoV-2 spike glycoprotein

**DOI:** 10.1016/j.jbc.2023.105014

**Published:** 2023-07-04

**Authors:** Samuel L. Freeman, A. Sofia F. Oliveira, Andrea E. Gallio, Annachiara Rosa, Maria K. Simitakou, Christopher J. Arthur, Adrian J. Mulholland, Peter Cherepanov, Emma L. Raven

**Affiliations:** 1 School of Chemistry, Cantock’s Close, University of Bristol, Bristol, United Kingdom; 2 Chromatin Structure and Mobile DNA Laboratory, The Francis Crick Institute, London, United Kingdom; 3 Department of Infectious Disease, St-Mary's Campus, Imperial College London, United Kingdom

**Keywords:** Heme, spike protein, SARS-CoV-2, biliverdin

## Abstract

The target for humoral immunity, SARS-CoV-2 spike glycoprotein, has become the focus of vaccine research and development. Previous work demonstrated that the N-terminal domain (NTD) of SARS-CoV-2 spike binds biliverdin—a product of heme catabolism—causing a strong allosteric effect on the activity of a subset of neutralizing antibodies. Herein, we show that the spike glycoprotein is also able to bind heme (*K*_D_ = 0.5 ± 0.2 μM). Molecular modeling indicated that the heme group fits well within the same pocket on the SARS-CoV-2 spike NTD. Lined by aromatic and hydrophobic residues (W104, V126, I129, F192, F194, I203, and L226), the pocket provides a suitable environment to stabilize the hydrophobic heme. Mutagenesis of N121 has a substantive effect on heme binding (*K*_D_ = 3000 ± 220 μM), confirming the pocket as a major heme binding location of the viral glycoprotein. Coupled oxidation experiments in the presence of ascorbate indicated that the SARS-CoV-2 glycoprotein can catalyze the slow conversion of heme to biliverdin. The heme trapping and oxidation activities of the spike may allow the virus to reduce levels of free heme during infection to facilitate evasion of the adaptive and innate immunity.

Coronaviruses use homotrimeric spike glycoproteins to bind cellular receptors to orchestrate entry into host cells by promoting the fusion of viral and cellular membranes. The SARS-CoV-2 spike glycoprotein protomer consists of two subunits, S1 and S2. SARS-CoV-2 spike binds to the angiotensin-converting enzyme 2 (ACE2), which maps specifically to the C-terminal domain of the S1 subunit, known as the receptor binding domain (RBD). The RBD is the major target of neutralizing antibodies, and adaptive mutations in viral variants have been localized in this region. The structure of the SARS-CoV-2 spike protein as well as the nature of its binding to ACE2 are well characterized (1, 2, 3).

The function of the N-terminal domain (NTD) of the S1 subunit is currently unclear. It has recently been established (4) that biliverdin binds to the NTD of the SARS-CoV-2 spike protein with high affinity (*K*_D_) in the nanomolar range (4)). Biliverdin is a green tetrapyrrolic molecule and a product of the degradation of heme in cells. Preparations of the SARS-CoV-2 trimeric spike protein and the S1 protein have been reported to be noticeably green in color (4, 5). Cryo-EM structures of the trimeric SARS-CoV-2 spike protein (at 3.35–3.5 Å) shows biliverdin binding in a deep hydrophobic pocket in each of three NTD domains of the protein (Fig. 1*A*, (4, 5)). An X-ray structure of the isolated NTD domain revealed details of the hydrophobic binding pocket and the binding orientation of the biliverdin metabolite at 1.8 Å resolution (Fig. 1*B*). Unidentified density in the same region in other published structures of SARS-Cov-2 (6, 7, 8, 9, 10, 11, 12) indicates that biliverdin was also present (at least at partial occupancy). Biliverdin binding substantially increased thermostability of the isolated NTD and restricted availability of a conformational epitopes on the spike NTD and decreased neutralization activity of a subset of antibodies targeting this domain (4, 13, 14). Another example of viral escape from recognition by antibodies through binding a metabolite was recently described for Norovirus (15).

**Figure 1:**
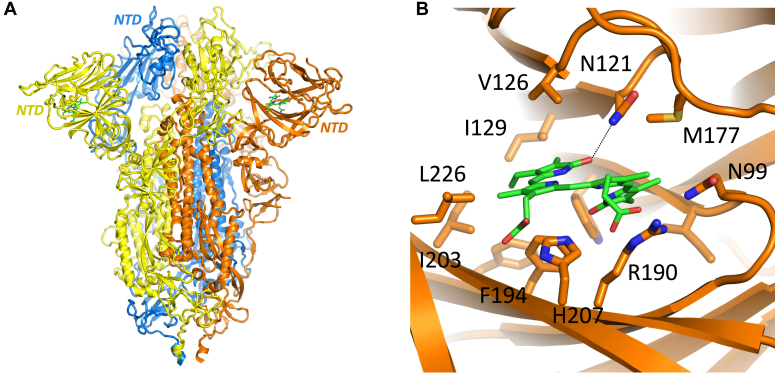
**Structures of SARS-CoV-2 spike protein.** *A*, cryo-EM structure (PDB 7NT9) of the trimeric SARS-CoV-2 protein (3RBD-down, in the closed conformation) in complex with the alpha isomer of biliverdin (biliverdin α, in *green*). The three protomers of the spike protein, forming the trimer, are coloured in *blue*, *orange*, and *yellow*. *B*, cryo-EM structure (PDB 7NT9) showing an expanded view of the biliverdin binding region of the N-terminal domain. Residues in close proximity to the biliverdin molecule are labeled, and the *dashed lin*e from N121 shows an important hydrogen bonding interaction with the tetrapyrrole (pyrrole D).

Biliverdin is produced in cells by heme oxygenase, which catalyzes the O_2_-dependent degradation of the tetrapyrrole heme (which is a red/brown color) to biliverdin (green) (16, 17). Carbon monoxide (CO) is produced as a by-product of the heme degradation process. Heme oxygenase thus influences not only the concentrations of free heme and biliverdin but also that of CO. This is particularly important considering that both heme and CO are important signaling molecules in cells (18, 19, 20, 21, 22, 23, 24, 25, 26). In this context, a number of connections between SARS-CoV-2 infection and heme concentrations have been made in literature. Notably, this includes the disease being defined as hemolytic, leading to a release of free heme into the blood and lung serum, as well as the downregulation of heme oxygenase-1 (27, 28, 29).

It is possible therefore, that the observation of biliverdin binding to the SARS-CoV-2 spike might be linked to the wider question of heme homeostatasis in COVID-19 and the involvement of heme oxygenase in controlling free heme levels. In this work, we have thus examined the interactions of the S1 subunit of the SARS-CoV-2 spike protein with heme.

## Results

### Heme binding to SARS-CoV-2 S1 protein

To establish the strength and nature of heme binding to the spike, we carried out heme titrations, according to previously published protocols (30). Heme binds most tightly to S1 from the prototypic (Wuhan-Hu-1) SARS CoV-2 strain characterized in 2019, with a *K*_D_ of 0.5 ± 0.2 μM (Table 1 and Fig. S1); the observed *K*_D_ is in the range typically observed for a weak exchangeable heme binding interaction in proteins (31, 32). S1 proteins from Alpha (B1.1.7, also known as Kent variant) and Delta (B1.617.2, Indian variant) SARS-CoV-2 isolates behaved similarly, binding biliverdin with a *K*_D_ of 1.4 ± 0.6 μM and 3.0 ± 1.6 μM, respectively (Table 1). The amino acid substitutions present in these variants are distant from the biliverdin binding pocket, Fig. S2, and the crucial residues in the biliverdin binding pocket of the NTD are conserved in all prevalent SARS-CoV-2 variants. Therefore, large differences in heme binding affinity would not be expected and indeed are not observed in Table 1.

**Table 1:** Binding constants for the interaction of heme with the S1 protein and site-directed mutants of the wild type S1 protein (H207A, N121Q, R190K), natural variants (Delta (B1.617.2, Indian variant) and Alpha (B1.1.7, Kent variant)), and the isolated NTD and RDB domains of the S1 protein

Sample	*K*_D_ (μM)
Wild type (Wuhan-Hu-1)	0.5 ± 0.2
RBD	70 ± 21
NTD	1.8 ± 0.9
H207A	0.6 ± 0.3
R190K	3.0 ± 1.2
N121Q	3000 ± 220
Delta (B1.617.2)	3.0 ± 1.6
Alpha (B1.1.7)	1.4 ± 0.6

Heme binding experiments of the isolated RBD and NTD domains of wild-type prototypic spike confirm significantly tighter heme binding to the NTD domain (*K*_D_ = 1.8 ± 0.9 μM, Table 1 and Fig. S1) than to the RBD domain (*K*_D_ = 70 ± 21 μM, Table 1 and Fig. S1); this confirms that the heme-binding interaction with the S1 protein occurs *via* the NTD (but non-specific, weak binding to RBD cannot be excluded). We also examined heme binding for various site-directed variants of the Wuhan-Hu-1 S1 NTD in the region of the biliverdin binding site (H207A, N121Q, R190K, Table 1 and Fig. S1) (4). Only the N121Q mutation, in which the asparagine residue in position 121 was replaced by glutamine, had a substantive effect on heme binding affinity (*K*_D_ = 3000 ± 220 μM). The likely causes of this dramatic effect of heme binding are examined below.

**Figure 2:**
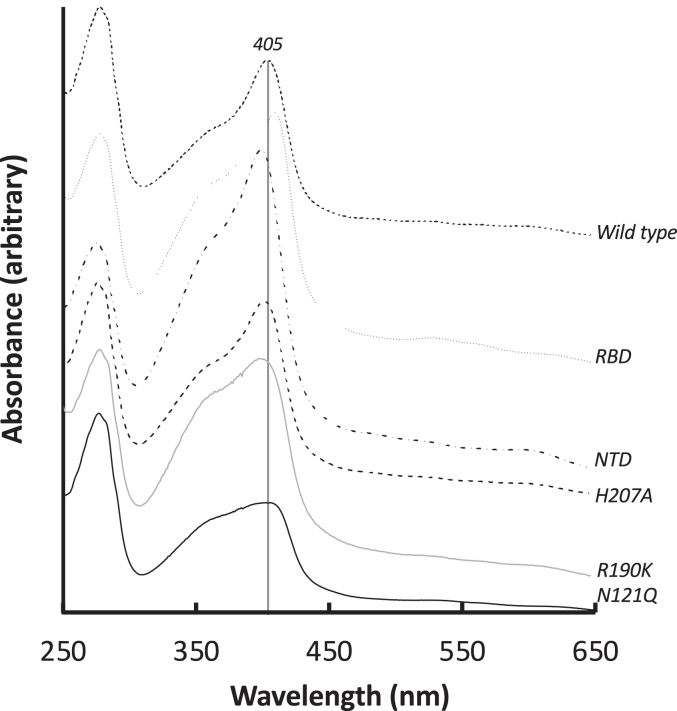
**Spectra of heme-saturated samples of the Wuhan-Hu-1 S1 protein ([heme]:[protein] = 1:1).** From top: Wild-type (*small dashes*), RBD (*dots*), NTD (*dot-dash*), H207A (*large dashes*), R190K (*solid grey*), and N121Q (*solid black*). Wavelength maxima for each protein are indicated in Fig. S1. All samples have been normalized at 280 nm for easier comparison.

Comparison of the shape of the heme-bound spectra for the S1 protein and variants, Figure 2, is informative. For the wild-type prototypic SARS-CoV-2 S1 protein and the isolated NTD, there is a clearly identified peak (λ_max_ = 405 and 402 nm, respectively, Fig. S1), with shape and wavelength maxima indicative of specific heme binding. This is not substantially altered in the H207A variant. On the other hand, there is a notable shift in both the shape of the spectrum and the wavelength maximum in samples which exhibit weak heme binding. Both the N121Q mutant (λ_max_ = 410 nm, Figs. 2 and S1*F*), and isolated RBD (λ_max_ = 413 nm, Figs. 2 and S1*B*) have Soret maxima shifted by ≈10 nm relative to wild-type S1 and NTD, suggesting a change in the nature of the heme ligation to the protein, which would be expected if the heme binding pocket in N121Q NTD is inaccessible. The R190K variant does not show a substantive shift in the Soret maximum, Figures 2 and S1*D*, although the shape of the spectrum is altered slightly; the binding affinity, Table 1, for this variant is only marginally changed.

### S1-catalyzed coupled oxidation of heme to biliverdin

**Figure 3:**
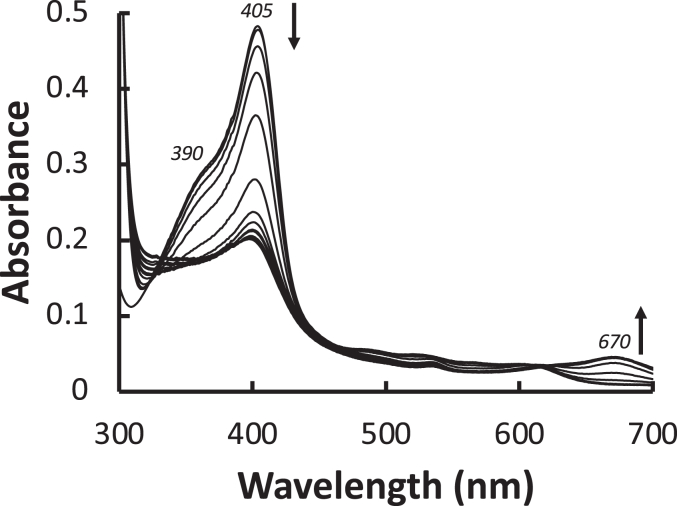
**UV-visible spectra showing the coupled oxidation process over a period of 5 h for the reaction of wild type S1 (2.5 μM) with ascorbate (0.5 mM) under aerobic conditions**. *Arrows* indicate direction of peak movements. Individual spectra were taken at 20-min intervals. After approximately 5 h, no further changes in the spectrum were observed, and the reaction is presumed to have reached completion. LC-MS analysis of the products of this reaction shows evidence of biliverdin formation (observed [M + H]^+^ = 583.2538 Da (2.1 ppm error), data not shown).

While heme oxygenase is responsible for the degradation of heme to bilverdin in cells, other heme-containing proteins, under suitable conditions, are capable of heme oxygenase-like activity. This process is referred to as coupled oxidation, and results in the slow formation of Fe(III)-biliverdin through the intermediate formation of α-hydroxyheme and verdoheme (as in the heme oxygenase reaction) (33, 34). Here we examined whether coupled oxidation of heme is observable in the heme-bound S1 protein. Figure 3*A* shows changes in the UV-vis spectrum of Wuhan-Hu-1 S1 which are very similar to those observed for the coupled oxidation process in other heme proteins (33). During the reaction, the Soret peak at 405 nm decreases in intensity and is blue-shifted. A broader peak appears concurrently centered at 670 nm, Figure 3*A*, similar to that previously reported during heme catabolism (33, 35, 36).

### Modeling

**Figure 4:**
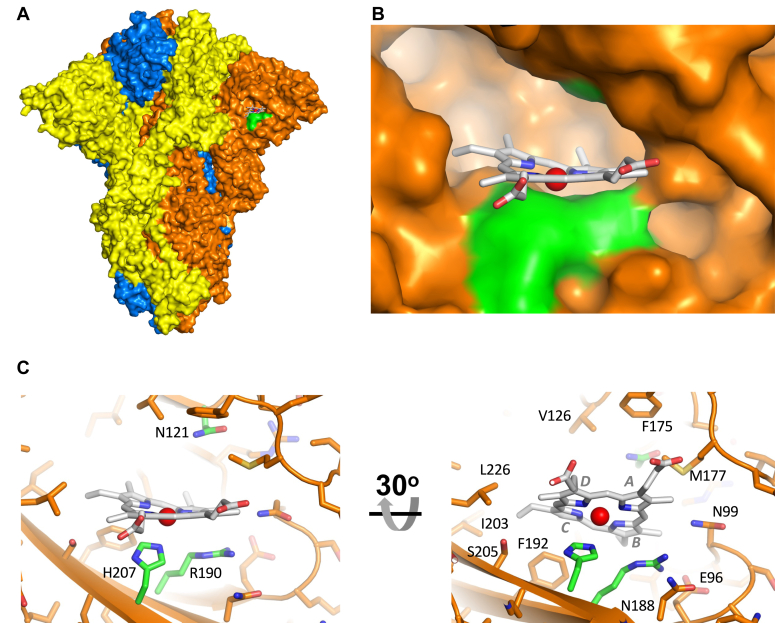
**Model of the heme-spike complex**. *A*, surface representation of the heme-binding pocket in the wild type S1. The three protomers are colored as in Figure 1*B*. Detailed view of the residues lining the proposed heme binding pocket. The *green color* highlights the location of N121, R190, and H207 (shown in the same orientation as in the *left panel* of (*C*)). The heme is colored *white* with the iron in the center as a red sphere. *C*, *Left*: Close up of the heme-binding pocket, with the N121, R190, and H207 residues shown in *green*, in the same orientation as in (*B*). *Right* – The same view as in (*A*) but with a 30° rotation about the *horizontal plane*, with other residues relevant to the discussion labeled. The heme pyrrole rings are labeled *A*–*D*.

Using the cryo-EM and crystal structures of the prototypic SARS-CoV-2 spike with biliverdin (pdb codes 7NT9 and 7B62), we constructed a model for the heme-binding interaction in the S1 subunit (Fig. 4). This model allowed us to identify relevant interactions involved in anchoring the heme to the protein and to understand the structural effect of the point mutations described above. The heme group fits well within the biliverdin binding cleft of the spike NTD (Fig. 4). The binding pocket is mainly lined by aromatic and hydrophobic residues (such as W104, V126, I129, F192, F194, I203, and L226, Fig. 4*C*), and it has the right shape and environment to accommodate and stabilise the hydrophobic heme. The heme pyrrole rings B and C are positioned deep inside the cleft whereas rings A and D together with the propionate residues point towards the solvent, as in all other heme proteins. In our model, the imidazole side chain of H207 is located near the heme group with the closest nitrogen atom (N^ε^) at a distance of ∼2.5 Å from the heme Fe atom. Curiously, removal of H207 (in H207A) has no significant effect on the heme binding, see above, which might be indicative of a weaker heme-histidine interaction than other histidine-ligated heme proteins.

In both the crystal structure of the NTD-biliverdin complex (PDB 7B62) and the cryo-EM structure of the ectodomain of the spike with biliverdin bound (PDB 7NT9) (4), the side chain of R190 is located near the heme rings A and B. This residue may help to stabilize heme binding by forming cation-π interactions with the pyrrole rings. Replacing arginine in position 190 with lysine (R190K) has a negligible effect on heme binding, which is not surprising given that both residues harbor a positively charged side chain able to interact with the heme pyrrole rings.

**Figure 5:**
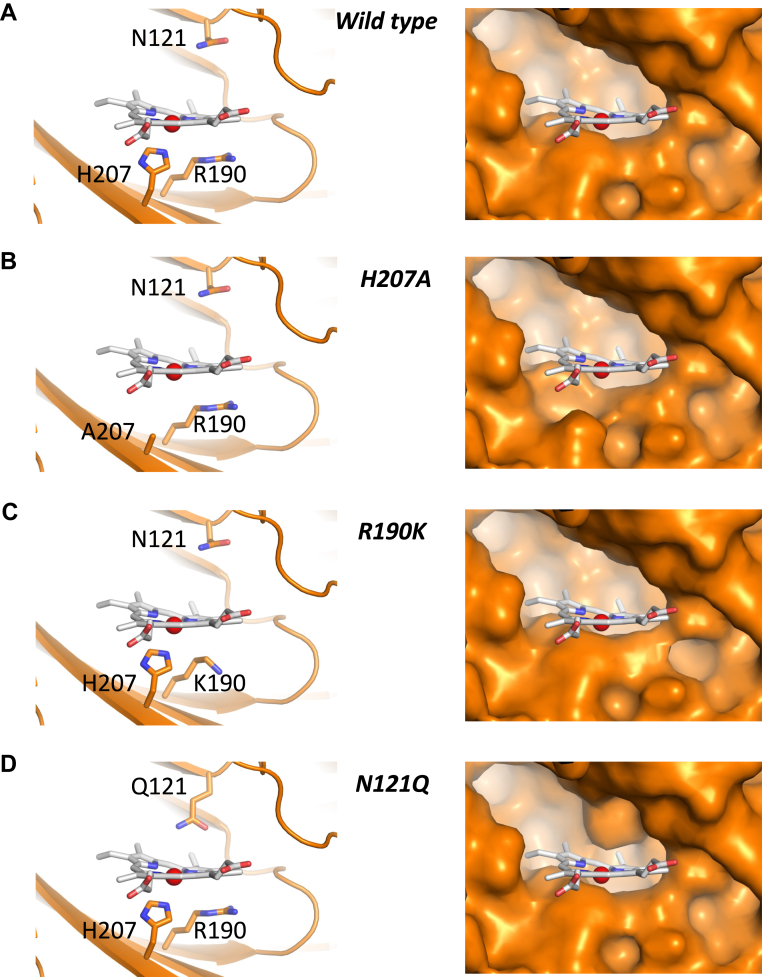
**Detailed view (*left*) and space-filling models (*right*) of the heme-binding pockets.** Figure shows heme pockets in (*A*) wild type, (*B*) H207A, (*C*) R190K, and (*D*) N121Q variants. The N121Q, R190K, and H207A mutations were introduced using Pymol. The heme is colored as in Figure 4. Note that according to the model, the substitution of N121 by a glutamine alters the shape and substantially reduces the total volume contained within the pocket, and thus reduces the space available for heme binding. There is other evidence from *in silico* analyses for heme binding at this same location (28, 46).

The most significant effect on heme binding was caused by the substitution of asparagine 121 for glutamine (N121Q). N121 lines the top part of the pocket and, according to our model, the effect of the N121Q mutation is largely a result of steric effects, Figure 5. Although asparagine and glutamine are both polar residues containing an amide group in their side chain, glutamine is one methylene longer. As can be seen from the model of the heme-N121Q S1 complex, Figure 5*D*, the side chain of glutamine 121 protrudes into the heme binding site, thus altering the shape and reducing the volume of the cleft and potentially introducing a steric clash with the heme in certain conformations. This would account for the changes in *K*_D_ for heme binding that we observe for this variant, Table 1. A similar mechanism likely accounts for the loss of biliverdin binding by N121Q S1(4).

Glycans cover the exterior of the spike and play crucial roles in shielding and infection (37, 38, 39, 40). Besides forming a disguise to evade the host’s immune system, glycans also modulate the dynamics of the important regions of the protein, such as the RBD and its binding to host receptors. We therefore compared our non-glycosylated spike:heme complex model with the fully glycosylated model for the closed wild-type spike built by Casalino *et al.* (37). This comparison, Fig. S3, identifies three *N-*glycans close to the heme/biliverdin binding site (at positions N122, N282 and N331). Given that these three glycans are located on the exterior of the protein and do not directly interact with the heme, they are unlikely to affect the binding interaction of heme with either the wild-type protein or the N121Q, R190K, and H207A variants (as all three residues are located inside the heme/biliverdin site).

**Figure 6:**
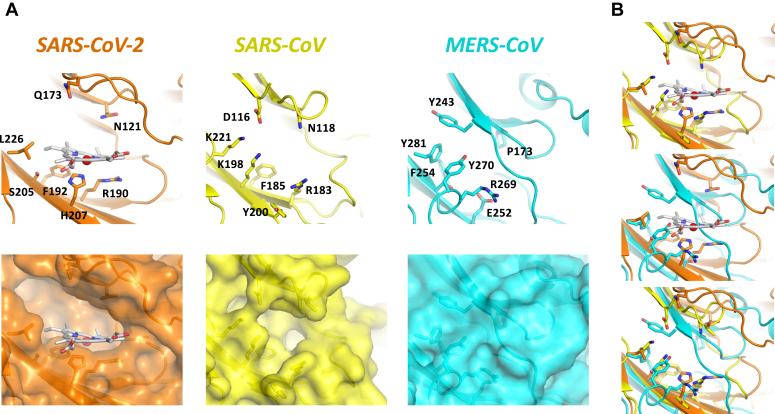
**Structural analysis of potential heme binding pockets in pathogenic coronaviruses.** (*A*) Comparison of the proposed heme binding pockets between SARS-CoV-2, SARS-CoV (PDB 5XLR (41)) and MERS-CoV (PDB 5X5C (42)). *B*, alignments of the heme-bound SARS-CoV-2 spike model (*orange*) with SARS-CoV (*yellow*) and MERS-CoV (*cyan*) structures. The *top panel* shows the superimposition of the heme-bound SARS-CoV-2 spike model with the cryo-EM SARS-CoV structure. The *middle panel* displays the superimposition of the heme-bound SARS-CoV-2 spike model with the cryo-EM MERS-CoV structure. The *lower panel* illustrates the overlapping of all three spike proteins. The heme group and some of the residues lining the heme-binding site are highlighted with *sticks*.

We then compared the heme pocket region in our model with other pathogenic coronaviruses, namely, SARS-CoV and Middle East respiratory syndrome coronavirus (MERS-CoV), Figure 6. Our model for the heme-S1 complex of SARS-COV-2 was aligned with cryo-EM structures of SARS-CoV (41) (PDB 5XLR (41)) and MERS-CoV (42) (PDB 5X5C (42)). As observed for SARS-CoV-2, in the SARS-CoV structure the proposed heme pocket is also present with several of the residues lining the pocket being conserved between both coronaviruses – namely N121 (N118 in SARS-CoV), R190 (R183) and F192 (F185), Figure 6. Histidine at position 207 in the SARS-CoV-2 protein is not conserved in SARS-CoV (Y270 in SARS-CoV). In SARS-CoV, the heme pocket is smaller than in SARS-CoV-2 largely as a result of the substitution of S205 and L226 in SARS-CoV-2 by two lysine residues (K198 and K221, Fig. 6). Such reduction in the volume of the proposed heme pocket would be consistent with the slightly lower affinity for biliverdin of SARS-CoV (*K*_D_ = 19.6 nM) compared to SARS-CoV-2 (*K*_D_ = 9.8 nM) (4). In the structure of the MERS-CoV spike protein, the equivalent pocket in the area of the proposed heme binding site is completely absent, with several bulkier aromatic residues (Y270, Y243, and F281) blocking its entrance. Additionally, in this MERS-CoV spike protein, the residues that most likely interact with the heme group, namely N121 and R190 in SARS-CoV-2, are substituted by P173 and E252, respectively. This may disfavor heme or biliverdin binding to the MERS-CoV protein, although to our knowledge heme binding to MERS-CoV has never been examined.

## Discussion

Heme and its metabolites—biliverdin, bilirubin, and carbon monoxide (CO)—play important roles in biology. In particular, CO is well-recognized as a signal transducer (23, 43). Being a strong metal-ligand that binds very tightly to heme iron, CO likely operates in concert with heme (18, 19), with heme itself implicated in signaling (18, 44).

Biliverdin is a by-product of heme catabolism: it is produced by the O_2_-dependent heme oxygenase enzyme, which converts heme, sequentially, to α-hydroxyheme, biliverdin, and CO. The identification of biliverdin bound to the SARS-CoV-2 spike glycoprotein (4) raises questions as to whether heme also binds in the same location, whether the biliverdin bound is a product formed from heme binding (as in heme oxygenase), and what, if any, advantages are thus conferred upon the virus.

To the first question, we have identified and characterized the binding of heme at low micromolar affinity to the NTD of the SARS-CoV-2 spike glycoprotein. This is weaker than the reported binding affinity for biliverdin (9.8 nM (4)), meaning that heme would have to be in excess to out-compete biliverdin, which may be the case if heme oxygenase is down-regulated (see below). We find that replacement of N121, which is involved in biliverdin binding, has a substantive effect on heme binding, which conclusively identifies this site as major heme binding location in the spike protein (Fig. 4). We cannot rule out other (weaker) binding sites for heme on the SARS-CoV-2 spike—this would be consistent with the known behavior of heme, as heme is a highly hydrophobic molecule and can sometimes bind non-specifically to proteins (45). *In silico* and surface plasmon resonance analyses support the existence of more than one heme binding site on the spike protein (28, 46, 47), one of which is focused around H207. Our analyses indicate that H207—which, like N121, is also located in the proposed heme binding pocket—has little effect on heme binding. Histidine is typically a strong heme ligand and is used extensively in many heme proteins. Our modeling indicates that the N^ε^ of H207 is within a reasonable distance (∼2.5 Å) of the iron but is further than the equivalent distance in, for example, myoglobin or the heme peroxidases (both typically (∼2.1 Å), where heme ligation by the N^ε^ of the histidine is highly robust. R190, similarly, has a very modest effect on heme binding. Examination of the SARS-CoV structure indicates that heme binding may also be feasible, based on the fact that the heme pockets are very similar (including the presence of the N121 equivalent, Fig. 6). In MERS-CoV, the equivalent binding pocket is absent.

To the second question—whether biliverdin is a by-product of an intrinsic heme oxygenase activity of the heme-bound SARS-CoV-2 protein—we have detected conversion of heme to biliverdin by the SARS-CoV-2 spike under *in vitro* (coupled oxidation) conditions. Numerous heme-containing proteins, under suitable conditions, exhibit coupled oxidation activity (33, 34, 48), although this is not necessarily a sign of *in vitro* heme oxygenase activity. Thus it is possible that biliverdin bound to SARS-CoV-2 spike may, at least in part, arise from the prior binding of heme and its catalytic conversion to biliverdin *in situ*. As shown here and elsewhere (4), both heme and biliverdin can bind to the viral spike independently. The ligands can be expected to occupy their shared binding site according to the respective affinities and availability.

The potential physiological roles of the heme binding and oxygenase activities of SARS-CoV-2 spike glycoprotein will require more scrutiny. However, heme metabolism and homeostasis are directly relevant to respiratory virus-caused pathology. For example, COVID-19 is known to be associated with extensive hemolysis (49, 50, 51) and elevated heme levels (29, 52). Moreover, hemoglobin levels can be decreased in COVID-19 patients (53, 54, 55, 56). Both of these symptoms may increase levels of free heme, which is a cytotoxic molecule. When not rapidly sequestered or metabolized, free heme can lead to inflammatory pathophysiology, a major part of acute respiratory distress syndrome (ARDS) (57). There are other indications that heme may be connected to COVID-19 infections—much of this implicates heme oxygenase, which has been suggested to play a role in the virus’ pathological impact (58, 59) and has been cited as a potential target for therapy in patients suffering from COVID-19 (60, 61, 62, 63, 64). The involvement of heme oxygenase is not unique to SARS-CoV-2, as decreased heme oxygenase expression has been documented in zika virus infections (65), influenza virus (66), HIV-1 (67), Dengue (68), and hepatitis B (69). A decrease in heme oxygenase activity would increase heme concentrations, lower CO concentrations, and could partially explain the clinical outcomes in COVID-19 patients (70). Note also that levels of indoleamine 2,3-dioxygenase (IDO-2), a tryptophan-metabolizing heme enzyme which, unlike IDO-1, is otherwise rarely expressed, are elevated in lung tissues of covid patients (71). Given the above context, it is possible that opportunistic binding of heme (or heme metabolites) could limit the structural rearrangements that are needed to facilitate antibody binding to the spike protein.

## Experimental procedures

### Expression and purification of SARS-CoV-2 S1 protein

S1 protein from the prototype SARS-CoV-2 strain Wuhan-Hu-1, including mutants (N121Q, H207A, and R190K) and individual domains (RBD and NTD) were produced by transfection of suspension-adapted human embryonic kidney 293 cells and purified as described previously (4). Wild-type S1 proteins from Delta (B1.617.2) and Alpha (B1.1.7) SARS-CoV-2 variants were produced following the same procedures. For the prototypic Wuhan-Hu-1 S1 protein, the RBD and NTD domains were also studied. SARS-CoV-2 S1 was depleted of biliverdin by exposing it to 0.5 M sodium acetate (pH 5.2) during size exclusion chromatography, as described previously (4).

### Electronic spectroscopy

All absorbance spectra were measured using a PerkinElmer Lambda 40 UV-visible spectrophotometer (25.0 °C). All measurements and titrations unless specified were carried out in HBSE buffer (150 mM NaCl, 1 mM EDTA, and 20 mM Hepes-NaOH, pH 8.0).

### Determination of heme binding constants

Heme binding constants were determined by absolute heme titrations, where measurements using a single cuvette containing protein and hemin were carried out, without attempting to subtract the free heme component. These data were then deconvoluted using a Multivariate Curve Resolution-Alternating Least Squares analysis (30). All data were deconvoluted according to a three-component system, yielding *K*_D_ values for heme binding. Small variations in *K*_D_ values are within the margin of error for this method and are likely caused by small traces of biliverdin present which could not be completely extracted during the protein purification process.

### Reaction of heme-bound SARS-CoV-2 S1 with ascorbate

A heme-bound solution of SARS-CoV-2 S1 was prepared by titrations as described above. An excess of *l*-ascorbic acid (0.5 mM) was then added to the hemin-bound protein and was allowed to react at 20.0 °C until no further spectral changes occurred, taking roughly 4 h. Spectral changes were monitored at wavelengths of 410 nm and 670 nm. When necessary, the sample was flash-frozen until required for further experimentation.

### Molecular modeling

The heme group was initially placed in positions analogous to those for biliverdin observed in the cryo-EM structure of the SARS-CoV-2 trimeric spike and crystal structure of isolated spike NTD (PDB entries 7NT9 and 7B62, respectively). Given that both experimental structures used as templates lack the complex glycosylation patterns observed in the wild-type protein, no glycans were added to our spike:heme complex.

The heme-bound structure was solvated in a single point charge (SPC (72)) water cubic box, considering a minimum distance between the protein and box walls of 10 Å. 150 mM sodium and chlorine ions were added to the box. The overall system contained a total of 646,237 atoms. The system was then relaxed by energy minimization with the GROMACS (73) software. The GROMOS 54A7 force field (74) was used to describe the protein, heme, and ions. An energy minimization, using the steepest-descent method in GROMACS (73), was performed to remove strain by performing 500 steps of minimization with harmonic restraints applied to all non-hydrogen atoms of the protein, followed by further 500 steps restraining the protein’s Cα atoms only, and finally by 500 steps without any restraints. The force constant used was 1000 kJ mol^-1^ nm^-1^. The mutagenesis menu, available in the Pymol software (75), was used to introduce the H207A, R190K, and N121K mutations. Molecular dynamics simulations were not performed.

## Data availability

All relevant data are available upon request from the corresponding authors.

## Supporting information

This article contains supporting information.

## Conflicts of interest

The authors declare that they have no conflicts of interest with the contents of this article.
