# Supplementary material for: Heme binding to the SARS-CoV-2 spike glycoprotein
Source: J Biol Chem. 2023 Jul 4;299(8):105014. doi: 10.1016/j.jbc.2023.105014 (PMC10416065; doi:10.1016/j.jbc.2023.105014)
Supplement: Supplementary figures [file mmc1.pdf]

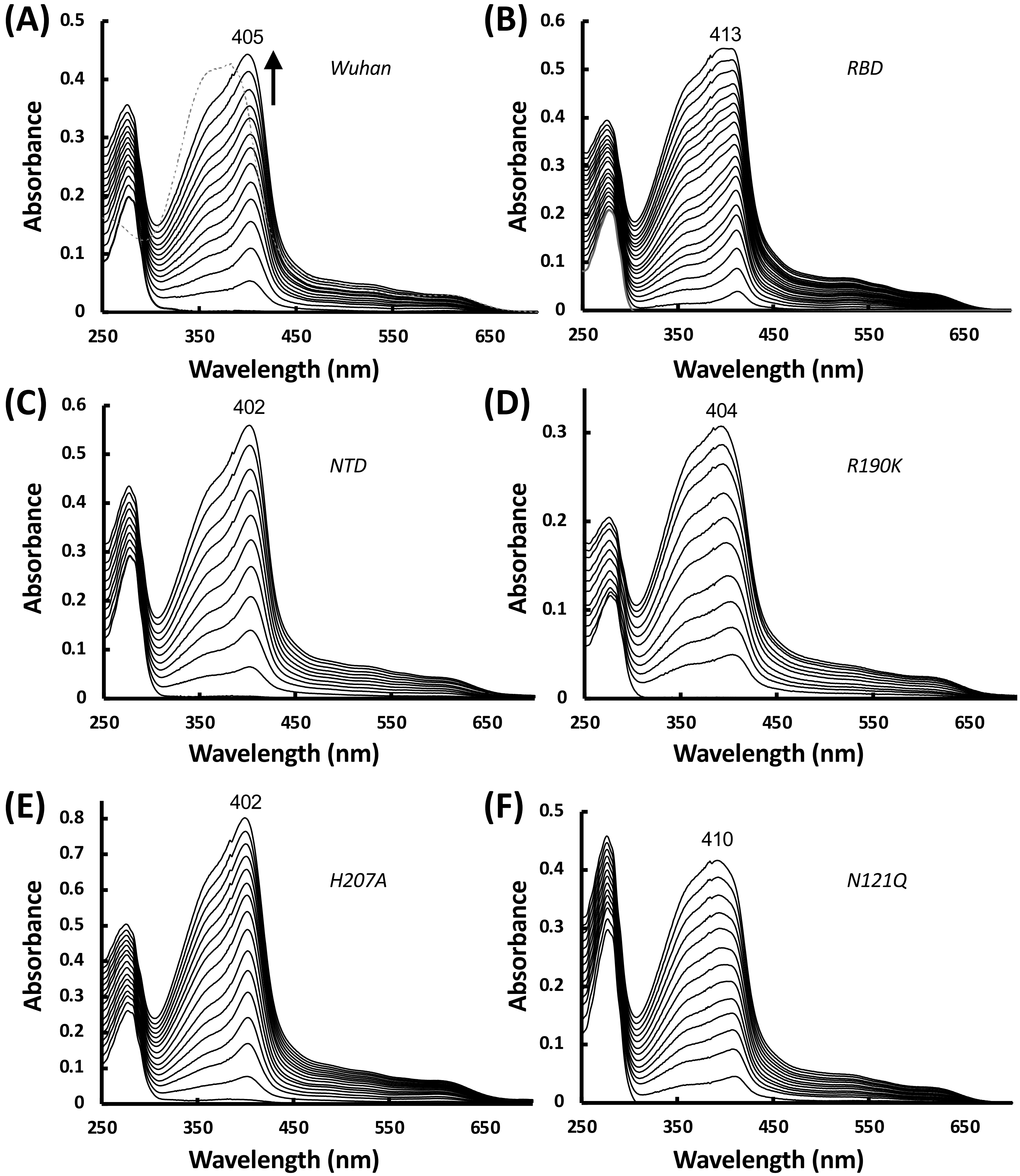

Figure S1. A series of UV-visible spectra showing titrations of heme into samples of the S1 protein: (A) Wuhan, (B) RBD domain, (C) NTD domain, (D) R190K mutant, (E) H207A mutant, (F) N121Q variant. The spectrum in (A) is annotated with an arrow to show the direction of intensity change for the Soret peak upon addition of hemin. A dashed grey line for the spectrum of free heme (at the beginning of the titration) is indicated for reference in Figure 2(A). The wavelength of the Soret band is indicated in each case (at a 1:1 molar ratio with heme).

(A) **(B)**

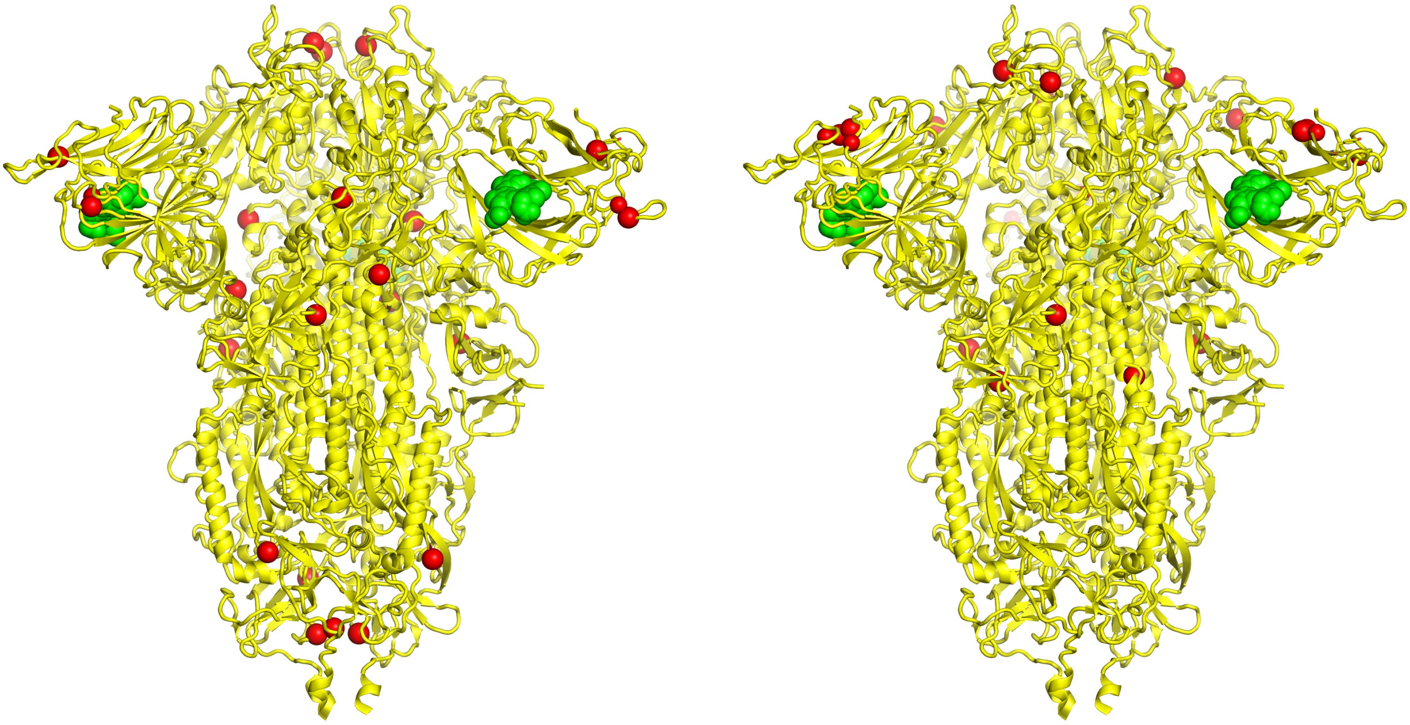

Alpha mutations ***Delta mutations***

***Figure S2*.** Alpha (**A**) and Delta (**B**) known mutations sites mapped onto the cryo-EM wild-type SARS-Cov2 spike structure (PDB 7NT9). The red spheres highlight the position of mutations and deletions whereas the green spheres represent biliverdin. The Alpha variant used in this paper contains seven spike mutations and three deletions, namely H69Δ, V70Δ, Y144Δ, N501Y, A570D, D614G, P681H, T716I, S982A, D1118H (outbreak.info SARS-CoV-2 Alpha VoC). Delta harbours seven mutations and two deletions in the spike, notably T19R, E156G, F157Δ, R158Δ, L452R, T478K, D614G, P681R and D950N (outbreak.info SARS-CoV-2 Delta VoC).

Figure S3 – Freeman *et al*.

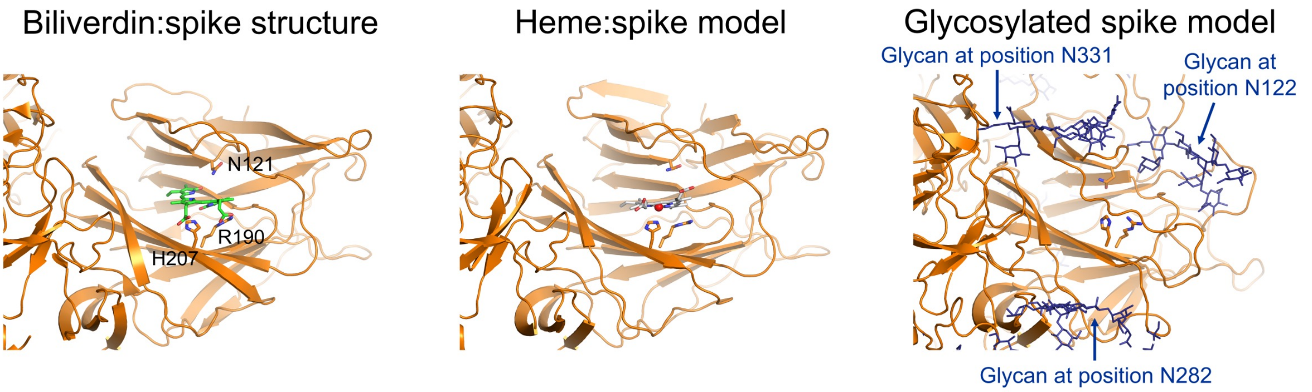

***Figure S3*.** Left: Cryo-EM structure of the SARS-CoV-2 wild-type spike (PDB 7NT9 (4)) showing a detailed view of the biliverdin site. Middle: The heme binding site in the non-glycosylated model of the heme-wild type spike complex modelled here. Right: Heme/biliverdin binding site in the model of the fully glycosylated wild-type spike in a closed conformation as shown by Casalino *et al. (37*), showing the glycans at positions N122, N282 and N331. Biliverdin (left) and heme (middle) are shown with green and grey sticks, respectively. Residues close to the biliverdin/heme molecule are labelled. In the right-hand figure, glycans are labelled and represented with dark blue sticks.
